# The impact of self-stigma in people with diagnosis of severe mental illness: a cross-sectional pilot study from a community psychiatry unit in Porto, Portugal

**DOI:** 10.1192/j.eurpsy.2023.1836

**Published:** 2023-07-19

**Authors:** A. S. Pinto, M. Almada, I. Fonseca, A. Sousa, A. Lopes

**Affiliations:** 1 ICBAS - Instituto de Ciências Biomédicos Abel Salazar, Universidade do Porto; 2 Unidade de Psiquiatria Comunitária, Serviço de Psiquiatria e Saúde Mental, Centro Hospitalar Universitário do Porto; 3 ICBAS; 4 EPIUnit and Instituto de Saúde Pública da Universidade do Porto (ISPUP); 5 Unidade Corino de Andrade, Centro Hospitalar do Universitário do Porto; 6 Unidade Corino de Andrade, Centro Hospitalar do Porto; 7 Serviço de Psiquiatria e Saúde Mental, Centro Hospitalar do Porto, Porto, Portugal

## Abstract

**Introduction:**

Self-stigma refers to the process in which a person internalizes negative stereotypes, beliefs, and prejudices about their mental illness, adopting a stigmatized view of themselves. Severe mental illness is one of the most socially exclusive stigmata and is associated with poor clinical and functional outcomes and social withdrawal.

**Objectives:**

In Portugal, investigation regarding self-stigma is scarce. In this study, we aim to evaluate the impact of self-stigma among people with diagnosis of severe mental illness (SMI). For this goal we assess the prevalence of self-stigma of psychiatric patients with diagnosis SMI; and investigate the correlates of elevated self-stigma levels.

**Methods:**

Fifty-one outpatients with SMI, were recruited from a community psychiatry unit from Porto, Portugal. After informed consent, evaluations included sociodemographic data, illness characteristics, and self-reported standardized scales. Self-stigma (ISMI), self-esteem (RSES) and quality of life (WHO-QoL) were assessed. Data analyses were performed using the SPSS version 28.0 (IBM Corp., Armonk, NY). *p*-values<0.05 were considered significant.

**Results:**

From the study sample, 66.7% were male, with mean age of 44.8±11.0 and 56.9% were single. 33.3% reported living with their parents while 31.4% were living with a partner/spouse. The majority of participants had a diagnosis of schizophrenia (60.8%). Concerning the level of education, 58.8% completed basic education, but most patients were retired due to illness (62.7%). In this study, moderate to high self-stigma levels was found in 31.4% participants. Proportion of elevated self-stigma was significantly higher in unemployed/retired patients *vs.* those who were active (39.0% vs. 0%; *P*=0.021). No significant correlations were found with age, level of education, age at diagnosis, duration of illness, and number of hospitalizations. In the correlations analysis, a negative correlation between self-stigma and self-esteem (rho=-0.745; P<0.001), as well as self-stigma and quality of life (rho=-0.585; P<0.001) was found. A positive relationship between self-esteem and quality of life (rho=0.551; P<0.001) was found.

**Conclusions:**

This study investigates, for the first time, the prevalence of self-stigma among outpatients with SMI in a community psychiatric unit from Porto. Our findings suggest a high prevalence of elevated levels of self-stigma among these patients. A significant association with being unemployed/retired was also found. Our results support previous evidence that internalized stigma is strongly associated with diminished self-esteem and impaired quality of life, in particular those aspects related to physical and psychological complaints. Targeting internalized stigma and self-esteem among patient with SMI will likely improve their quality of life, besides improving their clinical and functional outcomes.

**Disclosure of Interest:**

None Declared

